# CX3CL1 intracellular domain and adult neurogenesis

**DOI:** 10.18632/aging.102504

**Published:** 2019-11-29

**Authors:** Marc R. Benoit, Manoshi Gayen, Riqiang Yan

**Affiliations:** 1 Department of Neuroscience, University of Connecticut Health, Farmington, CT 06032, USA

**Keywords:** CX3CL1, fractalkine, adult neurogenesis, Alzheimer’s disease

It is widely accepted that stem cell niches in the subgranular zone of the hippocampus (SGZ) and subventricular zone (SVZ) exist in the adult mammalian brain and continually give rise to newborn neurons, referred to as adult neurogenesis [1]. There has been considerable work deducing the molecular mechanisms underlying neural stem cell proliferation and differentiation. Recently, adult neurogenesis is shown to be induced by a novel mechanism, which also ameliorates neurodegenerative phenotypes [2].

In the brain, CX3CL1, or fractalkine, is a type 1 transmembrane chemokine that has been widely shown to mediate neuron-microglia communication through the interaction with its cognate receptor, CX3CR1, exclusively expressed by microglia. For over two decades, attention has predominantly focused on the actions of the extracellular domain of CX3CL1, shed by α- secretases, and its role in inflammation. A new study by Fan et al. uncovers a novel mechanism by which a short intracellular C-terminal peptide of CX3CL1 (CX3CL1-ct), released after cleavages by α-, β- and γ-secretase, triggers back signaling to control neuronal survival and proliferation independent of CX3CR1.

Much like the Notch signaling pathway, CX3CL1-ct is translocated to the nucleus to induce large sets of gene expression. In order to elucidate the cellular effects of CX3CL1-ct, Fan et al. developed a transgenic mouse model expressing the CX3CL1 C-terminal fragment under the tetracycline inducible promoter, specifically induced in neurons when transgenic mice were crossed with mice expressing tetracycline-controlled trans-activator protein (tTA) by the CaMKIIα promoter. In the phenotypical analysis of CX3CL1-ct mice, a 30% and 40% increase in NeuN-positive granule cells of the dentate gyrus was noted when compared to wildtype littermates at three and six weeks old, respectively. BrdU labeling experiments confirmed enhanced neurogenesis in both the SGZ and SVZ. Since the transgene in this mouse model is inducible by doxycycline treatment, induction of adult neurogenesis by CX3CL1-ct was further explored. Indeed, the number of BrdU-positive neurons in the SGZ and SVZ was increased when the transgene is only expressed in the adult.

The extent to which promoting adult neurogenesis could be utilized for the treatment of neurodegenerative disease is unknown. Previous literature suggests that CX3CL1 plays a role in regulating amyloid deposition in the 5xFAD Alzheimer’s mouse model, presumably via its interaction with CX3CR1 on microglia [3]. Fan et al. chose to investigate the effect of neuronal CX3CL1-ct in the Alzheimer’s mouse model by crossing CX3CL1-ct and 5xFAD mice. Interestingly, they found a reduction in amyloid deposition in broad brain regions, including the subiculum and cortex, at 9 months of age. Additionally, neuronal loss normally seen in 5xFAD mice was abrogated with overexpression of CX3CL1-ct. These results uncover a novel mechanism by which a neuron-specific peptide, independent of microglial CX3CR1, can reduce amyloid beta deposition and subsequent neurodegeneration.

The nuclear localization of CX3CL1-ct and its neuroprotective effect in 5xFAD mice provides an intriguing question as to which downstream signaling pathways are activated. RNA sequencing analysis in CX3CL1-ct mice revealed over 200 significantly up-regulated genes known to control development, differentiation, transcription, and extracellular matrix signaling. Fan et al. further characterized the upregulation of the TGFβ2/3 pathway, demonstrating increased phosphorylation of downstream targets Smad2/3. It is known the TGFβ2/3-Smad2/3 pathway leads to the expression of many growth- and proliferation-inducing genes, however it remains unknown whether CX3CL1-ct increases the rate of neural progenitor cell division or increases survivability of newborn neurons.

**Figure 1:**
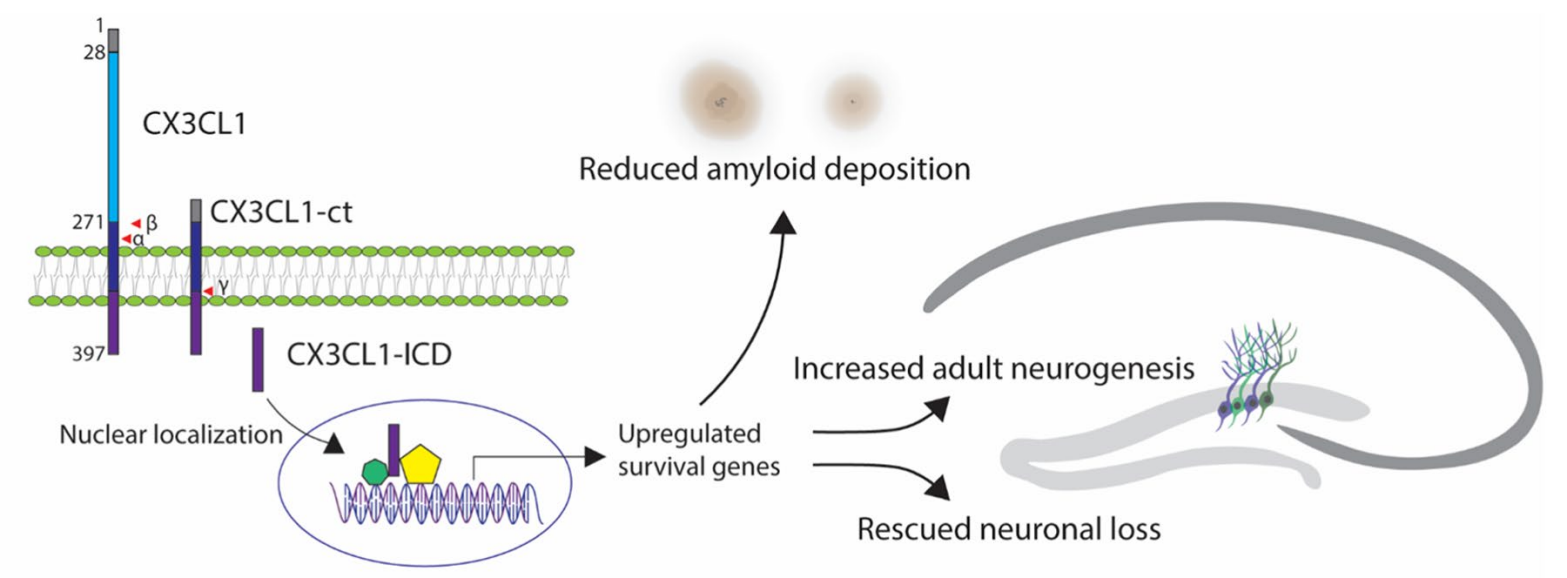
Full length CX3CL1 containing a signal peptide sequence (grey), extracellular C-X-X-X-C domain (blue), and C-terminal fragment (CX3CL1-ct), which includes transmembrane domain (dark blue) and a short intracellular domain (purple, CX3CL1-ICD), released after cleavages by α-, β- and γ-secretases. Nuclear localization of CX3CL1-ICD triggers gene expression that may induce upregulation of survival genes, leading to increased adult neurogenesis, rescued neuronal loss and reduced amyloid deposition in 5xFAD mouse models of Alzheimer’s disease.

Investigating CX3CL1/CX3CR1 signaling and its role in neurodegenerative disease has yielded inspiring but contrasting results. Deletion of CX3CR1 reduces amyloid deposition in mouse models overexpressing mutant APP [4], and prevents neuronal loss in the 3xTg mouse model overexpressing APP, PS1, and tau mutants [5]. This suggests that signaling microglia via CX3CR1 exacerbates amyloid pathology. Likewise, APPPS1 mice deficient in CX3CL1 have reduced amyloid deposition, however, hyperphoshorylated tau is increased [3]. The reduction in plaque formation and increase in tau phosphorylation cannot be reversed by reintroducing the soluble form of CX3CL1. Considering the results found by Fan et al., soluble CX3CL1, which lacks the intracellular domain, fails to induce a neuroprotective transcription program, likely explaining why soluble CX3CL1 provides no benefit (Figure 1).

Contrary to the protective role of disrupting the CX3CL1/CX3CR1 axis in amyloid deposition, microglia devoid of CX3CR1 exacerbates tau pathology in humanized tau mice, suggesting a protective role of the CX3CL1/CX3CR1 axis in the context of tauopathies [6]. Considering the nature of extracellular plaque clearance and intracellular neurofibrillary tangles, it is intriguing to think about the dual roles of CX3CL1 signaling to both microglia and neurons simultaneously. Future studies investigating the role of CX3CL1-ct in tauopathies will be interesting to pursue.

In summary, the finding of back-signaling from the intracellular domain of CX3CL1 is entirely novel and paves a way to re-think this signaling molecule for therapeutic application. Decreased neurogenesis has been observed in AD patients’ brains [7]. In the CNS, CX3CL1 is predominately expressed by neurons, and soluble CX3CL1 is reduced in the CSF of Alzheimer’s patients [8]. This finding offers a great premise for enhancing adult neurogenesis in AD brains, where neuronal loss is a pathological feature. Future studies examining how CX3CL1-ct induces a neuronal proliferative and survival program and its role as a potential therapy will be an exciting endeavor.
